# Retraction: Have We Substantially Underestimated the Impact of Improved Sanitation Coverage on Child Health? A Generalized Additive Model Panel Analysis of Global Data on Child Mortality and Malnutrition

**DOI:** 10.1371/journal.pone.0178903

**Published:** 2017-05-26

**Authors:** Paul R. Hunter, Annette Prüss-Ustün

The authors of the published article have identified issues with the analysis which raise concerns about the conclusions of this paper. When conducting the analysis, some of the country dummy variables were incorrectly generated in Excel prior to analysis in R. When the dummy variables are correctly generated, they substantially changed the shape of the partial residual plots such that the conclusions may no longer be valid.

In light of the concerns identified, the authors retract this publication.
